# *Burkholderia cepacia* complex: clinical course in cystic fibrosis patients

**DOI:** 10.1186/s12890-015-0148-2

**Published:** 2015-12-08

**Authors:** Tania Wrobel Folescu, Claudia Henrique da Costa, Renata Wrobel Folescu Cohen, Orlando Carlos da Conceição Neto, Rodolpho Mattos Albano, Elizabeth Andrade Marques

**Affiliations:** Department of Pediatric Pulmonology, Instituto Fernandes Figueira (Fundação Oswaldo Cruz, Ministério da Saúde), Av. Rui Barbosa, 716, 2nd floor, Flamengo, Zip Code: 22250-020 Rio de Janeiro, RJ Brazil; Department of Pulmonology, Universidade do Estado do Rio de Janeiro/UERJ, Rio de Janeiro, RJ Brazil; Department of Microbiology, Hospital Central da Aeronáutica, Rio de Janeiro, RJ Brazil; Department of Biochemistry, Universidade do Estado do Rio de Janeiro/UERJ, Rio de Janeiro, RJ Brazil; Department of Microbiology, Universidade do Estado do Rio de Janeiro/UERJ, Rio de Janeiro, RJ Brazil

**Keywords:** Cystic fibrosis, *Burkholderia cepacia* complex, Respiratory Function Tests, Body mass index

## Abstract

**Background:**

Pulmonary deterioration after *B.cepacia* complex (BCC) colonization has a heterogeneous pattern. The aim was to investigate the clinical outcome of BCC colonization in CF patients chronically colonized with *P. aeruginosa.*

**Methods:**

CF patients chronically colonized with *P. aeruginosa* were divided into three groups: intermittent (I), chronic (II) and no colonization (III) with BCC. Body mass index (BMI) percentile and spirometric parameters were analyzed at three different times in each group.

**Results:**

Fifty-six patients chronically colonized with *P. aeruginosa* were included. Of these, 27 also had evidence of BCC colonization (13 intermittent and 14 chronic). BMI percentile was significantly lower among patients chronically colonized by both *P. aeruginosa* and BCC. Mean values of FEV_1_ and FVC % were also significantly lower in these patients, both at the time of chronic BCC colonization and 24 months forward.

**Conclusions:**

Chronic BCC colonization is associated with significant loss of lung function. Lower BMI might be a risk factor for chronic BCC colonization, preceding these events.

## Key messages

- Chronic BCC colonization is associated with significant loss of lung function
- Lower BMI might be a risk factor for chronic BCC colonization

## Background

Respiratory disease is the major cause of morbidity and mortality in cystic fibrosis (CF) patients*.* The majority of CF patients develop chronic infection by *Pseudomonas aeruginosa*, which is associated with impaired lung function and decreased survival in CF [1]*.*

*Burkholderia cepacia* complex (BCC) is now recognized as a group of opportunistic pathogens in CF patients, usually associated with poor prognosis and patient-to-patient transmissibility. The exact pathophysiology of BCC colonization/infection remains unclear, and pulmonary deterioration has a heterogeneous pattern, leading to a fulminant development in almost 30 % of patients [2, 3].

Loss of lung function is still one of the main factor that contribute to mortality in CF [4]. Therefore, spirometric measurements – in particular, forced expiratory volume in 1 s (FEV_1_) % predicted and forced vital capacity (FVC) % predicted – are important surrogate measures of disease progression [4]_._ Nutritional status has a strong positive association with pulmonary function and survival in CF. Attainment of normal body mass index (BMI) is one of the major goals for CF treatment.

The aim of this study was to investigate the clinical outcome of BCC colonization in CF patients who were previously chronically colonized with *P. aeruginosa.*

## Methods

This is a retrospective study performed in two CF centres in Rio de Janeiro, Brazil: Instituto Fernandes Figueira (Fundação Oswaldo Cruz, Ministério da Saúde), for children/adolescents, and Hospital Universitário Pedro Ernesto – HUPE (Universidade do Estado do Rio de Janeiro – UERJ), for adults. Approval was obtained from the ethics committee on both institutions.

In both institutions, cultures of respiratory secretions were conducted in the Bacteriological Laboratory of HUPE/UERJ, according to standardized protocols established for CF patients, and were performed at least on a 3- monthly basis throughout the study [5]. The clinical samples were plated onto Sheep blood agar, MacConkey agar, Mannitol salt agar (Difco Labs, Detroit, MI, USA) and Burkholderia cepacia medium supplemented (Oxoid, Basingstoke, England). The microorganisms were characterized at the genus and species level by routine conventional physiological methods [6]. In order to identify the distinct genomovars, a 1043 bp PCR product corresponding to the recA gene was amplified by PCR. DNA sequence was performed in both directions with the PCR primers and the aid of two additional primers, BCR3 and BCR, as previously described [7, 8].

Patients’ inclusion criteria were: CF diagnosis according to *Cystic Fibrosis Foundation* consensus [9, 10]; regular clinical and laboratorial follow-up during the study period; chronic colonization with *P. aeruginosa* diagnosis (i.e., happened before the study period and was diagnosed when *P. aeruginosa* was isolated in more than 50 % of cultures of sputum or throat swabs taken in the previous 12-month period) [11]. Subjects who presented no respiratory colonization or solely *S. aureus* colonization were excluded from the study, as well as those who were being submitted to any kind of *P. aeruginosa* eradication protocol.

These patients (all chronically colonized with *P. aeruginosa*) were divided into the following groups:

- Group I – patients with intermittent colonization with BCC (1 or 2 isolates per year)
- Group II – patients with chronic colonization with BCC (3 or more isolates per year)
- Group III – patients never colonized with BCC

**Fig. 1:**
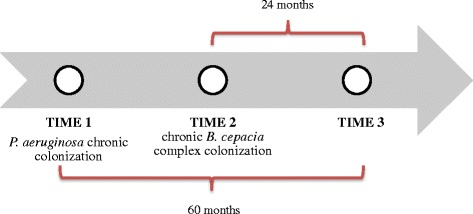
Timeline

For each patient, medical record data was evaluated, including gender, age, genotype and diagnostic criteria, state of pancreatic insufficiency (fecal fat or fecal elastase or need for exogenous replacement enzymes), CF-related diabetes (CFRD) and liver disease. The clinical outcome of each patient was also evaluated considering the following parameters: spirometric parameters such as forced expiratory volume in 1 s (FEV_1_) % predicted and forced vital capacity (FVC) % predicted, and body mass index (BMI) percentile for patients 2–19 y/o. These data were recorded for all groups during three different times (Fig. 1): Time 1: when patient was diagnosed as having *P. aeruginosa* chronic colonization; Time 2: when group II was diagnosed as having chronic colonization with BCC; since median time between the chronic colonization with *P. aeruginosa* and the chronic colonization with BCC was 3 years, this period of time was used to establish Time 2, for groups I and III; Time 3: all patients were also evaluated 5 years after the *P. aeruginosa* chronic colonization.

Descriptive statistical analyses were performed through construction of tables and charts. Non-parametric Mann–Whitney and Kruskal-Wallis tests were used to compare numerical and categorical variables with 2 or 3 categories respectively. The Wilcoxon test was used to analyze statistical significance of temporal variations within each group. The level of significance was set at probability (p) less than 5 % (p < 0.05).

## Results

From 2004 to 2013, 56 patients with chronic *P. aeruginosa* colonization were identified. Of these, 27 also had evidence of BCC colonization: 13 had intermittent colonization and 14 had chronic colonization. Clinical data is described in Table 1. Age of patients at the inclusion time ranged from 0 to 36 years old, most of them female (*n* = 38, 67.9 %); the majority were children or adolescents (*n* = 52; 92.9 %). Diabetes mellitus (CFRD) and liver disease related to CF were uncommon (3.6 and 1.8 %, respectively), whereas pancreatic insufficiency was present in most of the population (94.6 %). Regarding chronic colonization, no patients were chronically colonized for *H. Influenzae, Achromobacter spp and Stenotrophomonas spp.* Chronic colonization with *S. aureus* was found in 84.6 % in group I, 28.5 % in group II and 92.6 % in group III. Furthermore, chronic colonization with methicillin-resistant *S. aureus* was found in 7.7 % in group I, 0 % in group II and 7.4 % in group III.

**Table 1:** Patient data

	Group I	Group II	Group III	*p*-value
Gender (n)				0.056^*^
Male	4	8	6	
Female	9	6	23	
Age (mean age at time 1, in years)	9.2	11.1	9.4	0.744^**^
Mean annual hospital admissions per year	1.2	0.7	0.5	0.601^**^

*Obtained by *Χ* ^2^ test

**Obtained by Kruskall-Wallis test; Level of significance: p-value < 0.05

The average number of hospital admissions per year was 0.7, with no statistical difference among the groups. At each hospital admission, patients received intravenous antibiotic treatment. All patients with new growths of BCC were treated with a regimen of 3 intravenous antibiotics (meropenem + ceftazidime + amykacin) for 2 weeks, followed by 3 consecutive months of nebulised tobramycin. If BCC is isolated after initial colonization, patients received another course of step I. For further CBC isolation, therapeutic management was discussed by the multidisciplinary team. Death occurred in 7 patients (2 in group I and 5 in group II). No patient died in group III during the 5 years of observation.

Spiromeric data (FEV_1_ % and FVC % predicted) and BMI percentile were evaluated for all groups in the three different times previously described. Spirometric data were obtained from 49 of the 56 patients involved in the study: 10 of 13 patients in group I, 13 of the 14 patients in group II and 26 of the 29 patients in group III. The limiting factors for spirometry performance were: age less than six years old (6 patients), and neurological problems (1 patient).

There was no statistically significant difference between the groups for FEV_1_ % predicted (p-value: 0.267) or FVC (p-value: 0.911) at the time of *P. aeruginosa* chronic infection. However, at this time, patients of group II (who would course with chronic colonization with BCC 3 years later) already presented BMI percentiles significantly lower (mean: 17.8) than the other groups (mean: 41.4 and 53.9), and p-value was 0.002 (Table 2).

**Table 2:** Espirometric and BMI percentile data

		TIME 1			TIME 2			TIME 3		
Parameters	Groups	Mean values	Standard deviation	*P*-value	Mean values	Standard deviation	*P*-value	Mean values	Standard deviation	*P*-value
FEV_1_ %	I	78.9	27.0	0.267	82.9	27.9	0.176	86.6	22.3	0.004
	II	70.6	17.2		66.4	22.4		52.3	20.1	
	III	81.5	22.2		72.6	22.3		67.0	18.8	
	Total	78.2	21.8		73.1	23.7		66.7	22.5	
FVC %	I	85.5	24.2		88.9	23.4		93.6	18.7	0.032
	II	86.5	18.3	0.911	80.8	18.7	0.426	67.8	21.2	
	III	89.3	19.2		82.0	19.4		78.0	16.7	
	Total	87.9	19.4		83.1	19.9		78.1	19.9	
BMI percentile	I	41.4	25.4		43.5	26.9		45.4	20.8	0.035
	II	17.8	19.5	0.002	22.3	20.5	0.082	21.1	15.0	
	III	53.9	30.3		45.7	29.2		45.6	28.4	
	Total	42.2	30.6		39.8	28.1		39.8	26.1	

Group I: patients with intemittent colonization with *B. cepacia* complex bacteria (1 or 2 isolates per year)

Group II: patients with chronic colonization with *B. cepacia* complex bacteria (≥3 isolates per year)

Group III: patients never colonized with *B. cepacia* complex bacteria

FEV_1_ %: forced expiratory volume in 1 s % predicted

FVC %: forced vital capacity % predicted

BMI percentile: body mass index percentile

*p*-value obtained by Wilcoxon test (level of significance: p-value < 0.05)

Three years later, when patients from group II were diagnosed with chronic infection by BCC, mean values of FEV_1_ % predicted, FVC % predicted and BMI percentile were lower in group II, without statistical significance (*p*-value >0.05) (Table 2).

Five years later, mean values of FEV_1_ % predicted and FVC % predicted were significantly lower in patients chronically colonized with BCC (52.3 %; 67.8 %) when compared with those with intermittent colonization by BCC and those never colonized with BCC (p-values 0.004 and 0.032, respectively). Surprisingly, mean values of FEV_1_ % predicted and FVC % predicted for patients with intermittent BCC colonization were higher than those who were never colonized by BCC. At the end of the study (5 years after the *P. aeruginosa* chronic colonization), the mean value of the BMI percentile was significantly lower in patients with chronic BCC (21.1) than it was in groups I and III (45.4 and 45.6, respectively) with p-value 0.035 (Table 2).

Mean annual rates of decline for spirometric data (FEV_1_ and FVC % predicted) were statistically different between the groups. While group I (intermittent colonization with BCC) and III (never colonized with BCC) showed mean FEV_1_ % predicted annual rates of decline of 0 ± 2.9 % and −2.6 ± 2.9 % respectively, patients chronically colonized with BCC presented with a mean FEV1 % predicted annual rate of decline of −6 ± 6.0 %. When assessing FVC % predicted, mean annual rates of decline in intermittent or never colonized patients with BCC were 0.6 ± 2.5 % and −2.2 ± 2.9 %, respectively, while patients chronically colonized with BCC presented with −6 ± 7.9 % (Table 3).

**Table 3:** Spirometric data (Annual rate of decline: FEV_1_ % and FVC % predicted)

	Group	Number	Mean	Standard deviation	*P*-value Kruskall-Wallis
FEV_1_ %	I	10	0.0	2.9	0.002
	II	13	-6.0	6.6	
	III	26	-2.6	2.9	
	Total	49	-3.0	4.6	
FVC %	I	10	0.6	2.5	0.010
	II	13	-6.0	79	
	III	26	-2.2	2.9	
	Total	49	-2.6	5.2	

Number: number of patients who performed spirometry

Group I: patients with intermittent colonization with *B. cepacia* complex bacteria (1 or 2 isolates per year)

Group II: patients with chronic colonization with *B. cepacia* complex bacteria (≥3 isolates per year)

Group III: patients never colonized with *B. cepacia* complex bacteria

FEV_1_ %: forced expiratory volume in 1 s

FVC %: forced vital capacity

Level of significance *p*-value <0.05

In a subanalysis restricted to patients chronically colonized with *B. cepacia* complex bacteria, it was found that most of them had *B. cenocepacia* (*n* = 8) and *B. vietnamiensis* (*n* = 5). Analyzing spirometric data of FEV_1_ and FVC % predicted, there were no statistical differences between species in this group of patients. However, we noted a greater number of deaths in *B. cenocepacia* chronically colonized patients.

## Discussion

Previous studies have found that CF patients chronically colonized with BCC have a greater deterioration of lung function, require more frequent antibiotic therapy and also display increased mortality compared to patients colonized with *P. aeruginosa* [12, 13].

Although the clinical outcome after chronic *P. aeruginosa* colonization has been extensively analyzed and associated with deterioration of lung function, few studies have been done with chronic BCC colonization. McCloskey et al [14] assessed the impact of BCC infection in adult CF patients by measuring changes in pulmonary function and BMI in patients previously infected or never infected with *P. aeruginosa*. They concluded that infection with BCC results in a more rapid but variable lung function decline, which may be related to the strain involved. However, in all groups involved, there was no mention about chronicity of infection.

Correia et al [2] carried out a retrospective study of 31 patients with BCC infection who were categorized into two groups (I: intermittent isolations and II: chronic isolations). As expected, in the chronic isolation group, patients had higher mortality and number of hospitalizations and lower FEV_1_ values, in line with patients’ major deterioration. Some patients from the chronic isolation group had already exhibited deteriorated lung function before becoming infected with BCC, as a result of colonization with other pathogenic agents and disease progression. There was no comparative data between both groups regarding co-infection, lung function or BMI.

In our study, a higher number of deaths occurred in the BCC chronically colonized group. All patients in our study were previously diagnosed as having *P. aeruginosa* chronic infection. This aspect is relevant because the deterioration of any criteria in all groups wouldn’t be associated with *Pseudomonas aeruginosa*, a bacteria currently known to have impact on CF lung disease, and, therefore, eliminating this possible bias [4].

Patients who developed chronic colonization with BCC demonstrated significant lower FEV_1_ and FVC at this time, showing that *B. cepacia* complex chronic colonization might be a risk factor for lung volume decline. Higher mean values of FEV_1_ and FVC were found for patients with intermittent *B. cepacia* complex colonization. This might be related to a better patient and family adherence to treatment since first isolation of *B. cepacia* complex. The knowledge of the decrease in long-term survival and fear of progressive invasive bacteremic disease associated with BCC colonization might be responsible for this change in attitude regarding CF treatment.

The rate of decline in FEV_1_ % predicted has been studied in CF patients to better understand the progression of lung disease, in order to identify high-risk groups in whom aggressive therapy might be indicated and to assess therapeutic interventions. Risk factors associated with FEV_1_ decline include but are not limited to young age, high lung function, female gender, modifier genes, pancreatic insufficiency, poor nutritional status, viral respiratory infections, colonization with *P. aeruginosa* and BCC, and diabetes mellitus. The Epidemiologic Study of Cystic Fibrosis found that overall rates of FEV_1_ % predicted decline were −1.12, −2.39 and −2.34 % per year in 6–8-year-olds, 9–12-year-olds and 13–17-year-olds, respectively [15].

Frangolias et al [16], comparing two groups of adult CF patients (infected and not infected with BCC), found no significant differences in rates of FEV_1_ and FVC % predicted decline between cases and controls. In our study, mean rates of FEV_1_ % predicted decline were statistically different between the groups, and the group chronically colonized with BCC presented with a mean FEV_1_ % predicted decline of −6 % per year, which was much higher than expected in all ages. This shows the negative impact of chronic *BCC* infection in CF lung disease.

The association of better nutritional status with improvement of lung function is well documented, and poor nutrition is a risk factor for accelerated decline in lung function. However, it is not known whether BMI decline predates FEV_1_ decline. A retrospective study by Mc Phail et al [17] compared lung function and nutritional outcome in two CF birth cohorts. They found improvements in lung function and nutritional status in patients from ages 6–12, and a decreased rate of lung function decline associated with a higher baseline BMI (% predicted) and a slower rate of BMI decline. In our study, the BCC chronically colonized group had a lower baseline BMI, which might be a risk factor for chronic BCC colonization and disease progression.

Many studies have shown that in pulmonary colonization with *B. cepacia* complex, the outcome varies considerably, from a rapid fatal decline in lung function and bacteremia to an acceleration of pulmonary function decline or to chronic asymptomatic infection, suggesting that pathogenicity within *B. cepacia* complex varies [12]. In our study, *B. cenocepacia* was more frequent in the chronically colonized group (8/14). In this group, the number of deaths was higher and associated with *B. cenocepacia* colonization in 80 % of cases (4/5). *B. vietnamiensis* was isolated in one patient of group II who died (1/5). Mean time of death after chronic colonization with BCC was 5.2 years. Considering only the four patients with *B. cenocepacia* colonization, this time was reduced to 4.7 years.

Understanding functional consequences of CFTR mutations is important not only for population screening but also CF for management and treatment. McManus et al [18] performed genotype analysis on 59 adult CF patients and evaluated its correlation to *P. aeruginosa* and BCC chronic infection. They concluded that patients homozygous or heterozygous for the deltaF508 deletion are more likely to suffer airway colonization with BCC or *P. aeruginosa.* In our study, CF genotype was not available for all patients. Further evaluation of CF genotype will be useful to understand correlation of CF genotype and BCC colonization.

The retrospective nature of the study and the strict inclusion criteria limited the sample size. However, there are few studies contemplating BCC infection involving children and adults with CF.

## Conclusion

In conclusion, this study suggests that in patients previously identified as chronically colonized with *P. aeruginosa*, chronic BCC colonization is associated with lower spirometric data. However, lower BMI might preceed spirometric effects of BCC, possibly being a risk factor for chronic colonization.

## Abbreviations

BCC: *Burkholderia cepacia* complex
BMI: Body mass index
CF: cystic fibrosis
CFRD: CF-related diabetes
FEV_1_: forced expiratory volume in 1 s
FVC: forced vital capacity
HUPE: Hospital Universitário Pedro Ernesto
UERJ: Universidade do Estado do Rio de Janeiro
